# A multimenu system based on the P300 component as a time saving procedure for communication with a brain-computer interface

**DOI:** 10.3389/fnins.2013.00039

**Published:** 2013-03-25

**Authors:** Joanna Jarmolowska, Marcello M. Turconi, Pierpaolo Busan, Jie Mei, Piero P. Battaglini

**Affiliations:** ^1^Department of Life Sciences, B.R.A.I.N. Center for Neuroscience, University of TriesteTrieste, Italy; ^2^Department of Mathematics and Computer Science, Valparaiso UniversityValparaiso, IN, USA

**Keywords:** brain-computer interface, electroencephalography, P300, spelling, semantic organization

## Abstract

The present study investigates a Brain-Computer Interface (BCI) spelling procedure based on the P300 evoked potential. It uses a small matrix of words arranged in a tree-shaped organization (“multimenu”), and allows the user to build phrases one word at a time, instead of letter by letter. Experiments were performed in two sessions on a group of seven healthy volunteers. In the former, the “multimenu” was tested with a total of 60 choices: 30 “externally-imposed” selections and 30 “free-choice” selections. In the latter, 3 × 3 matrices were compared with 6 × 6 matrices. Each matrix was composed of letters or words, for a total of four matrices. Differences in classifier accuracy, bit rate and amplitude of the evoked P300 were evaluated. Average accuracy in all subjects was 87% with no differences between the selection methods. The 3 × 3 “multimenu” obtained the same level of classifier accuracy as the 6 × 6 matrices, even with a significantly lower amplitude of the P300. Bit rate was increased when using the 3 × 3 matrices compared to the 6 × 6 ones. The “multimenu” system was equally effective, but faster than conventional, letter-based matrices. By improving the speed of communication, this method can be of help to patients with severe difficulties in communication.

## Introduction

Every year thousands of individuals lose control of their motor pathways. This may be due to cerebral stroke or to the progression of a neurodegenerative disease (e.g., Flynn et al., 2008; Wijesekera and Leigh, 2009). When damage is particularly severe, even communication can be compromised (Birbaumer et al., 1999; Kunst, 2004).

In order to re-establish the possibility to communicate, a Brain-Computer Interface (BCI) has been proposed as a promising tool (Farwell and Donchin, 1988; Birbaumer et al., 1999; Pfurtscheller et al., 2003). Generally, a BCI is a device that accepts commands contained in neurophysiological signals to interact with the external world without using explicit motor output pathways such as nerves and muscles (Wolpaw et al., 2002). This allows individuals to create new communication channels. Neurophysiological signals can be recorded from external electrodes by using electroencephalography (EEG). The software translates those brain signals into actions that can drive different types of devices. Users of such systems learn to control various types of endogenous brain patterns, such as slow cortical potentials (SCP; Birbaumer, 2006) and sensorimotor rhythms (SMR; Pfurtscheller et al., 2000, 2003; Wolpaw et al., 2002), or to force a sensory stimulation that elicits a characteristic EEG pattern referred to as an event-related potential (ERP; Farwell and Donchin, 1988). In fact, ERPs relate to a brain activity that is elicited in response to external or internal events. Many BCI systems are based on visually-evoked potentials and, particularly, on the P300 wave, which is a late ERP attentional component (Krusienski and Wolpaw, 2009). In the BCI system, the P300 response is most frequently elicited with the so-called “oddball paradigm” (Fabiani et al., 1987; Sellers et al., 2006a). In this paradigm, the subject is presented with a sequence of events that can be classified into two categories: frequent and infrequent, which are either unexpected or expected. Events, also called stimuli, in the infrequent category elicit an ERP characterized by the P300 component; the less probable is the infrequent stimulus, while the greater is the amplitude of the P300 component that is evoked (Farwell and Donchin, 1988).

In a typical application, the use of the P300 component enables the user to sequentially select alphanumeric characters from a keyboard-like matrix on a personal computer screen by focusing attention to the desired target for several seconds (Farwell and Donchin, 1988). This is known as “P300 Speller,” and evokes the P300 response by briefly highlighting each row and column of a certain matrix in a random order. Following an oddball paradigm, each sequence contains rare targets and frequent non-target stimuli (Sellers et al., 2006a). The item that the user focuses his/her attention upon can be recognized by classifying the brain potentials that are evoked by the flashing of each row/column, and making an intersection between the row and the column that elicited a P300 response. Until a few years ago, this system required a relatively long time for the selection of a single target. It has been reported that the P300 BCI can usually provide between 3 and 8 selections per min (Ryan et al., 2011). Since a less time-consuming procedure is obviously desirable, in recent years these systems of communication have been improved upon. For example, Brunner et al. (2011) demonstrated that the use of a classical P300 Speller paradigm with more invasive methods, such as electrocorticography (ECoG), can greatly decrease the communication time, thereby strongly increasing the amount of information transferred by the system in 1 min (bit rate). Those authors showed that it was possible to sustain a rate of 17 characters/min (allowing a communication transfer rate of 69 bits/min). Other not invasive studies have investigated the effect of the stimulus used to elicit evoked potentials (ERPs) by using for example faces (Kaufmann et al., 2011, 2012a). Increased responses to familiar faces and improved performance were observed, thanks to a significant reduction of stimulus sequences that were needed for the correct classification of characters. A predictive spelling system has recently been proposed wherein the time to complete the target phrase was shorter compared to the conventional P300 Speller (Ryan et al., 2011). However, despite the improvement in overall output in this new system, accuracy was significantly higher in the classical paradigm (Ryan et al., 2011). Differently from the predictive paradigm by Ryan et al. (2011), accuracy did not decrease with the paradigm proposed by Kaufmann et al. (2012b). In this study it has been proposed a new integration of predictive text directly into the matrix of the targets. The results show benefit in terms of bit rate and accuracy obtained by the participants. Other studies have shown that even single trial classification is possible to achieve faster and more efficient control of external devices (Piccione et al., 2006). In addition, even the classical Farwell and Donchin's speller system (Farwell and Donchin, 1988) has been modified, for example, by filling the matrix with icons and other elements to best fit it with the task that the user had to perform. Such was the case for painting, (Münssinger et al., 2010), surfing the web (Mugler et al., 2010), or even remotely control an external device in a pure domotic context (Wang et al., 2005). In all these cases, it has been shown that there is essentially no difference between the response evoked by icons and that evoked by letters or numbers. It has also been shown that in the classical P300 Speller system, a smaller matrix size and shorter inter-stimulus interval (ISI) between the flashes of rows and columns are optimal to increase the effectiveness of BCI (Sellers et al., 2006b). Moreover, a 6 × 6 matrix has a higher bit rate compared to a 3 × 3 matrix. This demonstrates that matrix size and ISI are both important variables to optimize a BCI system (Sellers et al., 2006b). Finally, it has also been proposed to enhance non-invasive approaches for communicative BCI thanks to the utilization of different brain signals, such as for example by using the Steady-State Visual Evoked Potentials (SSVEP; Cecotti, 2010; Volosyak, 2011) or by the introduction of speller approaches based on motor imagery (e.g., Scherer et al., 2004).

In this regard, we propose a “multimenu” system as a tool not only to increase communication speed, but also to provide the possibility to select as many deliverable messages as possible. The cue is to use words that represent wishes and needs, but also general topics that could be hardly represented by icons or images alone.

The first goal of the present study is to demonstrate that the “multimenu” is a valid and easy-to-learn BCI communication tool that is faster than conventional spellers, given that the dictionary of semantically-related words is adequate.

The second goal is to validate the validity of the “multimenu” BCI system by comparing it with a classical P300 Speller in terms of classifier accuracy, bit rate and signals amplitude. We assumed that the amplitude of the P300 in the “multimenu” will decrease with respect to the classical P300 speller system, because of the smaller matrix size, and according to the lowest ratio between attended and unattended items (e.g., Duncan-Johnson and Donchin, 1977, 1982). However, despite the lower amplitude of the P300 component in the “multimenu” system, we hypothesized that the “multimenu” will, however, allow reliable performance in terms of accuracy. Finally, we hypothesized that the “multimenu” will produce at least the same level of bit rate of the classic P300 Speller. This will allow the user to communicate better with fewer selections (of words instead of letters), with an evident gain in terms of time needed to produce an informative message.

## Materials and methods

### Participants

Seven healthy adults were recruited (2 males, 5 females, age range 22–31 years, mean age 23.9 years, standard deviation 3.2). All were naive to BCI use and had full comprehension and use of the Italian language. The study protocol was prepared in accordance with the Declaration of Helsinki. Each subject signed informed written consent before the experiment that could be left at any time. The ethical committee of the University of Trieste approved the study protocol. All subjects participated in two experimental sessions performed on different days.

### Structure of the “multimenu” system and control conditions

**Figure 1:**
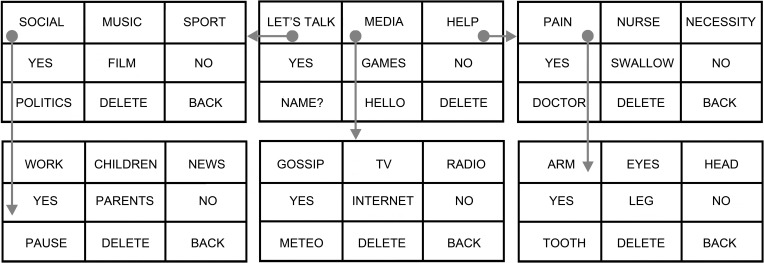
**Structure of the “multimenu.”** Top center: main menu. All other inserts: first and second level submenus. Gray circles are examples of selections; arrows indicate the semantically related submenus. In each level, there is the possibility to delete an incorrect selection with the “Delete” command and to return to the preceding level with a “Back” command. To speed up communication, “direct-output” words were also included, allowing, for instance, to write “hello,” the name of the partner, or quick answers such as “yes” or “no.”

The “multimenu” system is based on matrices containing nine Italian words arranged into rows and columns (since the system is based on 3 × 3 matrices containing words, it can also be referred to as 3W). Each word had the size of 16% of the total width of a 15.4 inch screen that was placed at about 50 cm from the subject. Words allow the subject to navigate into a tree-shaped series of submenus, where the first selection does not lead to a direct output sentence, but rather is a link for a second level of the “multimenu” (Figure 1). This level contains words that can be used to compose a sentence and that may give access to further levels. In each level, there is the possibility to delete a wrong selection and to return to the previous level using dedicated commands. To speed up communication, “direct-output” words were also included in the menus (especially in the first), allowing, for instance, to write “hello,” the user's name or to produce quick answers such as “yes” or “no,” by performing a single selection.

In the first on line study, carried out to demonstrate the robustness of the “multimenu” system for comparison purposes and to reduce variability related to the choices of participants, the target phrases or words to be completed were dictated by the experimenter (“externally imposed”—EI—mode), or freely chosen by the participants themselves (“free choice”—FC—mode). Each subject completed a total of 60 selections, equally randomized between FC and EI modes.

In the P300 study, four kinds of matrices were used, namely 3 × 3 and 6 × 6 grids words (named 3W and 6W, respectively) and 3 × 3 and 6 × 6 grids of letters (named 3L and 6L, respectively), forming an adapted version of the classical P300 Speller. In each condition, all the elements of the grids were always present on the screen (i.e., nine elements for 3L and 3W, 36 elements for 6L and 6W). In the word matrices, items were composed of different numbers of letters (i.e., from 2 to 9 letters, average word length of 5 in the 3W matrix and from 2 to 7 letters, average word length 4 in the 6W matrix). Different word lengths were used to account for the different size of matrices. For each condition, subjects were requested to select the same elements (for a total of 20 target items per condition) in order to maintain a reliable comparison among users. Selections covered all the rows and columns contained in the matrix to reduce the influence position on the results. Finally, under 3W and 3L conditions, some words were used more than once compared to the other conditions to fulfill the 20 items selection.

### EEG data acquisition and pre-processing

**Figure 2:**
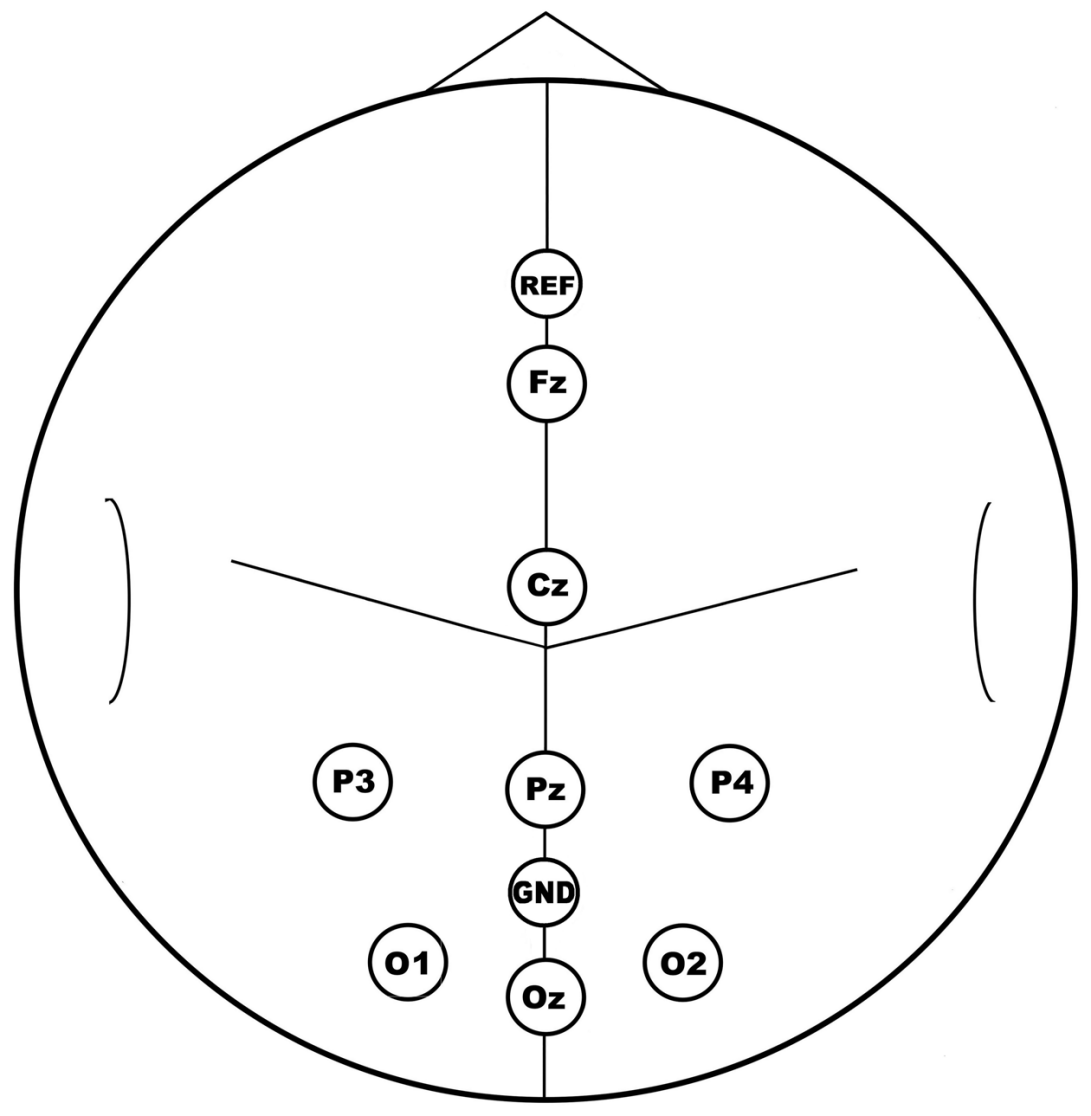
**Location of electrodes.** Electrode (channels) position is displayed on a simplified head model where the main sulci are also outlined. REF, reference electrode (Afz); GND, ground electrode (Poz).

EEG was recorded with a standard cap (Electro-Cap International, Inc.) where electrodes were placed following an adapted version of the EEG 10–20 coordinate system (e.g., Jurcak et al., 2007). Signals were recorded from eight channels (electrodes Fz, Cz, P3, Pz, P4, O1, Oz, O2; Figure 2) that were referenced to the Afz electrode and grounded to Poz. The final signal was obtained by applying a common average reference. The same channels were used for classification. Impedance was always maintained below 5 kΩ. Signals were amplified and digitalized with a Micromed amplifier (SAM 32FO fc1; Micromed S.p.A., Italy; high-pass analogical filtering 0.1 Hz; sampling rate frequency 128 Hz). A general-purpose and free BCI software platform, BCI2000 (Schalk et al., 2004; http://www.bci2000.org/), controlled stimulus menu presentation, data collection and online processing. From each channel, an 800 ms data segment, related to each row/column flashing (see later), and representing the epoch of interest for that stimulus, was extracted and analyzed by the software.

### Calibration session

An initial session was conducted in copy mode to detect the channel-specific features needed to optimize the P300 response and train the P300 classifier of the BCI software. This initial session was conducted by using the “P300 Speller” matrix (6 rows × 6 columns). In this initial session, the alphanumeric string was displayed at the top of the monitor with the next item-to-spell (target “item” is intended as the attended stimuli and refers to the letter or character the users is counting the flashes of) indicated in brackets at the end of the string itself. For example, if the assigned string was “CIAO” (“hello”), at the beginning of the run the word CIAO appeared, followed by [C]. The task was to wait and count the number of times the target item flashed on the screen and then report it during the pause after target item presentation. Counting helped the user to focus on the item itself, and the same strategy was used in all experiments. After each target item was presented, a pause of 2 s was allowed. This procedure was repeated until the string of items was completed. Subjects performed the initial session with a flash duration and an ISI between two consecutive flashes of 125 ms. Flashes were organized into sequences, where one sequence is referred to a single intensification of each row and of each column contained in the matrix. In the case of 6 × 6 matrix, a sequence was composed of 12 intensifications. A total of 20 sequences was used for each item, for a total of 40 flashes for every target item (the amount of target item intensifications corresponded to the number of sequences multiplied by 2), in addition to a total of 200 flashes for the non-target ones. As a consequence, each selection took about 1 min, and about 4 min to complete a run (a “run” is intended as the string of items to be completed), also considering pre/post run and pre/post item selection pauses. Thus, for each participant, about 20 min of data were recorded to complete the five runs. These data were then used to determine the presence of the P300 component and to train the feature weight classifier of the BCI2000 software.

### Classification

**Figure 3:**
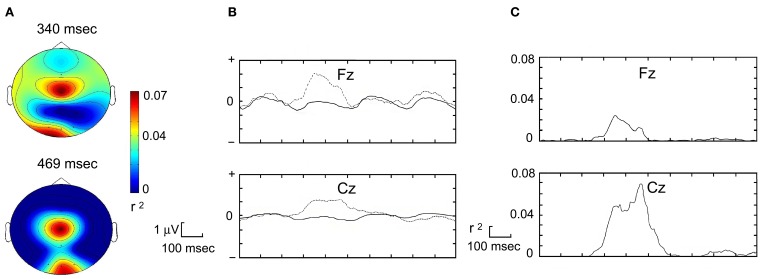
**Different aspects of the P300 component, recorded in a prototypical subject. (A)** Topography plots for the attended item in two different time frames. R-squared scale on the right of the insert. **(B)** The time courses at the Cz and at the Fz electrodes of the voltages for stimuli including (dashed line) or not including (continuous line) the target stimuli. **(C)** Corresponding R-squared time course that indicates a statistical difference between the target and non-target stimuli.

As suggested by Krusienski et al. (2008), the target item is located at the intersection of the row and column that elicits the P300 response. Stepwise linear discriminant analysis (Donchin et al., 2000; Krusienski et al., 2006) was used to determine its presence and estimate the classification coefficients of target and non-target items. P300 amplitude and its spatial and temporal localization were evaluated. As a result of this discrimination process, the P300 classifier created a transition matrix that described the probability that a defined response was effectively recognized by the classifier. The diagonal of this matrix represented the classifier accuracy (Kronegg et al., 2005). Offline analysis tools available in the BCI2000 software were used for the related aspects of the P300 component (Figure 3). Moreover, the P300 classifier tool created a user-specific table containing all these information, which were inserted in the user's parameters to have optimal on-line classifications and ensure high accuracy selections.

### Experiment I: online spelling accuracy

The purpose of this experiment was to assess the robustness of the “multimenu” system by testing performance accuracy over a defined number of selections in an online (free spelling) mode of BCI2000, where the user received a feedback after every selection. Accuracy was examined on a total of 60 selections, equally divided between FC and EI modes. In both modes, accuracy was calculated as the number of correct selections made by the participant and dividing this value by the total number of selections (i.e., 30 for each condition in this session): in particular, in the FC mode, the user was asked to report, in the pauses between one selection and the next, the item he/she would like to select in the next trial. In this way, it was possible to have online, continuous monitoring of the progress of the experiment and the level of accuracy.

### Experiment II: accuracy of classifier, bit rates and P300 amplitude

A second experiment was performed to better define the accuracy levels obtained in the previous one and it was conducted in a copy mode of BCI2000 (one in which the user has to spell a predefined text displayed on the screen). Data from the four sets of stimuli (3 × 3 and 6 × 6 matrices of words—3W and 6W, respectively—, and 3 × 3 and 6 × 6 matrices of letters—3L and 6L, respectively) were crossed to create four conditions and check the existence of significant differences between them. The P300 amplitude was the dependent variable and was analyzed for each condition. Further dependent measures were the classification accuracy (calculated referring to the classification coefficients of the four conditions), the bit rate and the amount of transferred bits during one selection. Thus, during the calibration sessions, in this second experiment, participants were provided with strings of target items (intended as alphanumeric symbols in 3L and 6L conditions, or words in 3W and 6W ones) to select according to the calibration protocol already described. To avoid the possible effect of fatigue, the different matrices were presented in a balanced fashion among conditions. Four different sessions were made, one for each condition, and data were then analyzed with the offline analysis tools mentioned above. In this second experiment, a total of 15 sequences was applied. For the selection of each target item, 180 flashes for the 6 × 6 matrices and 90 flashes for the 3 × 3 matrices were needed. The desired target item was contained in 1/6 and 1/3 of highlighted rows and columns in the case of the 6 × 6 and 3 × 3 matrices, respectively. Timing of selection in all conditions is reported in Table 1.

**Table 1:** **Time needed for selection of target items**.

**Multimenu**		**P300 speller**	
**Selection time (sec)**	**Selection/min**	**Selection time (sec)**	**Selection/min**
22.5	2.6	45	1.3

Values are obtained multiplying the inter stimulus interval and the stimulus duration (125 ms for both) by the number of rows and columns and number of sequences (n = 15).

For each of the four conditions, a total of 20 target items was selected. In the 6 × 6 conditions (6L and 6W), a total of 3600 flashes (target and non-target) was performed. In the 3 × 3 conditions (3L and 3W), a total of 1800 flashes (target and non-target) was presented. Data from 600 target items (3 × 3 and 6 × 6 matrices) were compared with those obtained from 1200 (3 × 3 matrices) to 3000 (6 × 6 matrices) non-target items for each session. Classifier accuracy and bit rate were computed for each condition by using the BCI2000 toolbox “P300 classifier.” Once the classifier accuracy was checked, the amount of bit transferred in one selection was calculated following the definition proposed by Wolpaw and colleagues (Wolpaw et al., 1998) for noisy channels (Shannon and Weaver, 1949) using the formula:

$$\text{Bit trans}={\text{log}}_{2}\text{N}+\text{P}\times {\text{log}}_{2}\text{P}+(1-\text{P})\times {\text{log}}_{2}(1-\text{P})/\text{N}-1$$

where “N” is the number of the possible choices present in the matrix, “P” is the classifier accuracy and, consequently, “1-P” is the classification error.

Once this value was found, bit rate, which corresponds to the amount of transferred bits in 1 min (Wolpaw et al., 1998), was calculated by relating it to V (where V is the application speed in trials/second, i.e., how many items are recognized per second) according to the formula:

Bit rate=V×Bit trans

The amplitude of P300 was defined as the highest mean amplitude that was visible on single electrodes compared to baseline in each subject and condition. Absolute values were used for this calculation. P300 was identified and scored as the highest peak appearing only in the rare condition, and normally comprised in an interval between 200 and 500 ms post-stimulus. The highest values were always obtained from Fz, Cz, or O1 electrodes.

### Statistical analysis

Statistical analysis was firstly conducted on data obtained from the “multimenu” system to examine its accuracy. More specifically, we compared the user's performance (over a total of 60 selections) of the FC selection method vs. the EI one. Analysis was carried out using a non-parametric statistic (Wilcoxon test), considering that, in this case, data were not normally distributed. We also accounted for the presence of many observations that were numerically similar. As a consequence, an “exact” Wilcoxon test was used.

Successively, data obtained from experiment II were analyzed to evaluate the existence of significant differences between the amplitude of the P300 component when different matrix sizes and types of stimuli were presented. In this case, data were normally distributed and a repeated measures ANOVA was conducted considering the size of the matrix (3 × 3 or 6 × 6) and type of stimuli (letters or words) as main factors. When an effect representing a main factor or an interaction resulted significant, simplification of the model was conducted using a Student's *t*-test. Moreover, we compared the bit rate obtained for each of the four conditions indicated above. In this case, data were not normally distributed and an “exact” Wilcoxon test was applied, also accounting for the presence of observations with the same result. Statistical tests were always two-tailed. Normality of data was verified using the Shapiro–Wilk test. A *p* < 0.05 was considered statistically significant.

## Results

### Experiment I: accuracy of spelling

**Figure 4:**
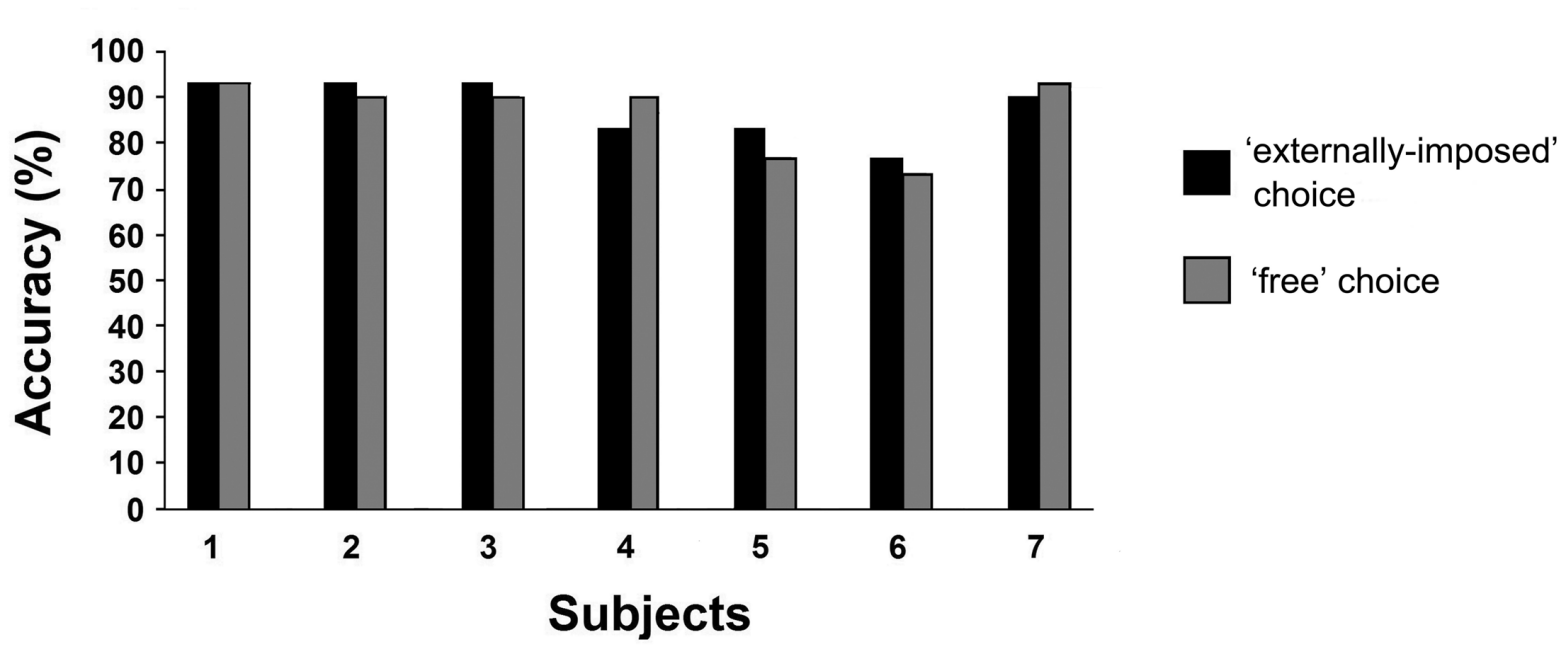
**Accuracy for all subjects.** Data collected from 30 selections made when the word to be chosen was suggested by the experimenter (EI mode: black) or by the subject (FC mode: gray).

In order to demonstrate the robustness of the “multimenu,” even compared to currently-available software, its accuracy was analyzed, for each user, over 30 EI and 30 FC selections. No significant differences were found between conditions (Wilcoxon test: *p* = 0.72). Mean accuracy in all subjects was 87.6% (SD 6.6) and 86.7% (SD 8.2) for the EI and FC conditions, respectively. Data are summarized in Figure 4.

### Experiment II: accuracy of classifier, bit rate and P300 amplitude

#### Accuracy of classifier

Mean classifier accuracy in the 3L condition was 97.14% (SD 0.03) and 97.86% (SD 0.04) in 3W condition. In the 6L and 6W conditions, classifier accuracy was 98.57% (SD 0.02; SD 0.04, respectively). Subject-by-subject data are reported in Table 2.

**Table 2:** **Classifier accuracy for all subjects with respect to the different matrix sizes and the different types of stimuli**.

**Subjects**	**3 × 3 letters**		**3 × 3 words**		**6 × 6 letters**		**6 × 6 words**	
	**Accuracy**	**Bit rate**	**Accuracy**	**Bit rate**	**Accuracy**	**Bit rate**	**Accuracy**	**Bit rate**
1	95%	7.09	100%	8.24	95%	6.06	100%	6.72
2	95%	7.09	100%	8.24	100%	6.72	100%	6.72
3	100%	8.24	95%	7.09	95%	6.06	100%	6.72
4	95%	7.09	100%	8.24	100%	6.72	100%	6.72
5	100%	8.24	100%	8.24	100%	6.72	100%	6.72
6	100%	8.24	90%	6.24	100%	6.72	90%	5.59
7	95%	7.09	100%	8.24	100%	6.72	100%	6.72
Mean	97.14%	7.56	97.86%	7.79	98.57%	6.53	98.57%	6.56
SD	(0.03)	(0.6)	(0.04)	(0.8)	(0.02)	(0.3)	(0.04)	(0.4)

Bit rate is also reported. The bottom rows show the mean and standard deviation (SD) for each condition.

#### Bits transferred during one selection and bit rate

Number of transmitted bits per selection in the 6 × 6 matrices at the probability level of 100% was 5.2, while it was 3.2 in the 3 × 3 matrices at the same level of probability. The probability level indicates the offline accuracy of target classification. Data relative to different levels of probability are reported in Table 3. The average bit rate for 3L condition was 7.56 (SD 0.6), and 7.79 (SD 0.8) for 3W condition, while in the 6L and 6W conditions, the average bit rate was 6.63 (SD 0.3) and 6.63 (SD 0.4), respectively. All data used for bit rate calculation are summarized in Tables 2 and 3.

**Table 3:** **Number of transmitted bits per trial in the different matrix sizes and different levels of probability (*P*)**.

**3 × 3**	**6 × 6**	**3 × 3**	**6 × 6**	**3 × 3**	**6 × 6**
***P* = 1**	***P* = 1**	***P* = 0.95**	***P* = 0.95**	***P* = 0.9**	***P* = 0.9**
3.2	5.2	2.7	4.6	2.4	4.2

Significant differences were found between bit rates obtained in the 3L vs. 6L conditions (Wilcoxon test: *p* = 0.016), and between those obtained in the 3W vs. 6W conditions (Wilcoxon test: *p* = 0.016).

#### P300 amplitude

**Figure 5:**
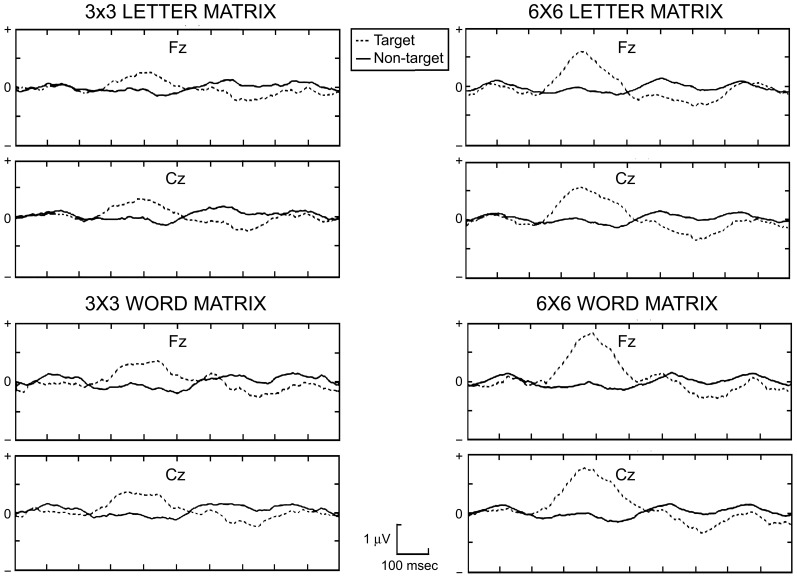
**Grand-averaged P300 responses for all participants in the 4 conditions (3L, 3W, 6L, 6W).** Plots were performed for two representative channels (Fz, Cz).

Figure 5 shows the comparison of P300 amplitudes in all conditions. For all items, the amplitude was clearly lower in the 3 × 3 matrices compared to the 6 × 6 ones. The effect related to the matrix size computed with ANOVA was significant [*F*_(1, 6)_ = 49.04, *p* < 0.0009]. No significant effect was evident when considering the main factor related to the use of letters or words [*F*_(1, 6)_ = 3.7, *p* = 0.1] or the interaction between main factors [*F*_(1, 6)_ = 0.78, *p* = 0.41]. Analyses conducted with Student's *t*-tests confirmed that the P300 amplitude obtained in the 3 × 3 conditions (independently of the type of stimulus used, i.e., letters or words) was significantly smaller in comparison to that obtained in the 6 × 6 condition [*t*_(6)_ = 6.94, *p* < 0.0009]. Data are summarized in Table 4 and Figure 5.

**Table 4:** **Mean P300 amplitudes (in μV; standard deviations in brackets) obtained for each condition**.

	**3 × 3 letters**	**3 × 3 words**	**6 × 6 letters**	**6 × 6 words**
Mean	0.80	0.90	1.34	1.62
SD	(0.27)	(0.14)	(0.35)	(0.53)

Data were calculated on absolute values for all subjects and were individuated as the highest P300 peak (normally from 200 to 500 ms post-stimulus on Fz, Cz, or O1 electrode).

## Discussion

In the present investigation, we present a BCI based communication procedure which relies on the use of small matrices of words organized in a hierarchical structure instead of a larger matrix of letters. We wanted to investigate if the use of this “multimenu” system increases the efficiency and speed of communication compared to a classical P300 Speller. For this purpose, two experiments were performed.

The primary goal of the study, obtained with the first experiment, was to demonstrate that the method was robust and effective for a communication system. As a matter of fact, a high level of accuracy was obtained in all subjects, independently of the selection method used (EI or FC), even when a substantial number of selections was performed. The number of selections that was used (*n* = 30) was adequate to communicate basic information and express feelings, desires and needs that the user is experiencing.

A second goal of this study was to test the efficiency of the “multimenu” system using the measures of P300 component, offline accuracy and bit rate in a BCI paradigm. The main hypotheses were that the “multimenu” should improve communication by selecting whole words instead of single letters and that, due to matrices size, an effect on waveform and component morphology should also be evident.

Indeed, we observed clear differences when considering matrix sizes. In fact, higher amplitudes were found with the 6 × 6 matrices, independently of the utilization of words or letters, probably because of the higher ratio between expected and unexpected items (e.g., Duncan-Johnson and Donchin, 1977; Allison and Pineda, 2003; Sellers et al., 2006b). Since the “multimenu” is a 3 × 3 matrix of words, it evoked lower P300 amplitudes compared to the classical speller system. On the other hand, relevant variations in the type of stimuli (letters or words) were not observed, suggesting that this parameter does not affect the identification of the response. Thus, even if lower P300 amplitudes were obtained from the “multimenu,” the online accuracy remained considerably high compared to the recommended threshold for reliable BCI (Kübler et al., 2001; Serby et al., 2005), and good performance was maintained in selections with this system.

As a consequence, due to its reduced selection time, the “multimenu” allows a higher bit rate than a conventional speller system, since it is possible to make more selections in 1 min. In fact, the number of transferred bits for each selection was higher in the 6 × 6 matrices compared to the 3 × 3 ones when using letters or words, while, on the other hand, the bit rate for the different matrix sizes and stimulus types showed that 3 × 3 matrices of the “multimenu” had a higher bit rate compared to 6 × 6 matrices of the P300 Speller. Even if a bias in this observation is present (see below a discussion on the limits of Wolpaw's definition), the higher bit rate in the “multimenu” allowed for faster transfer of information in a defined time range using 3 × 3 matrices compared to the 6 × 6 ones with the classical P300 Speller, allowing at the same time a meaningful communication.

These results suggest that a “multimenu” can provide a substantial advantage to an individual one communicating via the P300 component in an online environment, especially for expression of simple messages.

There is to say that other BCI studies are already known which showed improvements in terms of accuracy and/or bit rate with respect to classic P300 speller, often exceeding the bit rate obtained in the present study (Diez et al., 2011; Ryan et al., 2011; Kaufmann et al., 2012b). Kaufmann et al. (2012b) effectively demonstrated that the integration of predictive text directly into the matrix may result in higher benefits in terms of speed of spelling without loss of accuracy. Despite the lower levels of bit rate obtained in the present study with respect to the evidence in the literature, the strength of the present system is that it allows to save time with respect to a classical P300 Speller condition. In fact, the selection of a target can provide the immediate expression of a message or, in the case of two/three selections, it can lead to the expression of a whole sentence or concept. In the “multimenu” system, thanks to the dimensions of the matrices, the user obtains the feedback in half the time compared to the time required with the classic P300 Speller.

It has been theorized that performance of P300 based BCI should be related to the degree of the attention of users. Fazel-Rezai described that habituation can affect the P300 detection for real-time applications. In particular, the level of attention can decrease with repeated presentation of the same type of stimulus (Fazel-Rezai, 2009). The author suggested that habituation in the speller system can be reduced by altering the region of stimulus location on the screen, its background, and other visual effects that create a change in the paradigm. The novelty increases the user's attention for the presentation of new symbols and stimuli. In the present experiments, the level of attention might have been facilitated by the use of words, which change according to the intended meaning, and by the immediate feedback given by the system. Moreover, the changes of matrices from one submenu to another might allow the maintenance of a high level of attention, avoiding a habituation effect that can affect the P300 detection in experiments where the same matrix is always displayed (e.g., Fazel-Rezai, 2009). This hypothesis is also supported by the fact that classifier accuracy, which can be also seen as a value of the attention level, was very high for all the subjects. For the same reasons, the “multimenu” might reduce fatigue, which is one of the main causes of error in classical BCI speller programs (Fazel-Rezai and Ahmad, 2007), even if, in a series of different studies, the accuracy of the P300 signal has been found to be poorly affected either by fatigue or by performance (e.g., Sellers and Donchin, 2006).

The present results are also consistent with previous P300 studies (including P300 BCI research) which show that P300 amplitude is higher when the probability of appearance of the target item is lower, as in the present classic P300 Speller (e.g., Duncan-Johnson and Donchin, 1977; Allison and Pineda, 2003; Sellers et al., 2006b). The grand average of the P300 in Figure 5 shows that the target waveform differs in amplitude between the 3 × 3 and the 6 × 6 conditions. Most notably, when considering the target responses, the P300 peak was much larger in the 6 × 6 than in the 3 × 3 matrix, and was generally larger when considering electrode locations Fz and Cz. This particular distribution was likely related to the utilization of a common average reference. In fact, to the best of our knowledge, a classical P300 spatial distribution could be observed at the level of electrodes Fz, Cz, Pz (e.g., Krusienski et al., 2008; Duncan et al., 2009). Furthermore, a widely used electrode reference in P300 research is the earlobe or mastoids (e.g., Miller et al., 1991; Picton et al., 2000). Thus, the present spatial distribution of the P300 could be related to the particular experimental settings.

One limit of the present work relies on the limitations of Wolpaw's definition (Wolpaw et al., 1998), which has been used to calculate the bit rate. In fact, it has been reported that the utilization of Wolpaw's definition for calculation of the bit rate may give incorrect values in particular conditions and, thus, may require some assumptions such as those regarding the equiprobability of classes in desired and manifested selections (e.g., Kronegg et al., 2005; Spüler et al., 2012). It was also suggested that the Wolpaw's definition cannot be always properly used, since it is based on some assumptions that are not always completely absolved in common BCI speller experiments (e.g., Kronegg et al., 2005). In fact, it assumes that “N” symbols are recognized by the classifier only if “N” symbols are expressed by the user. Another assumption is that all symbols should have the same a priori occurrence probability. Finally, the classifier accuracy “P” should be the same for all target symbols, and the same should be true when considering the classification error “1-P” (equal distribution of wrong selections). Actually, in an on-line classification analysis, it is difficult to believe that these assumptions are always completely absolved, due to the unpredictable choices that a user can make during spelling. For example, some studies have shown that classifier accuracy may be different between presented symbols (Mason and Birch, 2000; Perelmouter and Birbaumer, 2000; Obermaier et al., 2001), and that the classification error is not equally distributed over them (Mason and Birch, 2000; Obermaier et al., 2001). Unfortunately, it is not easy to find a better and universal criterion that allows for comparison with the majority of the previous BCI experiments (e.g., Spüler et al., 2012). For this reason, we adopted the Wolpaw's definition, also considering that the majority of the previous BCI studies (e.g., Allison and Pineda, 2003; Sellers et al., 2006b; Krusienski et al., 2008) employed this approach, thus allowing to better compare the present results with the existing BCI literature.

A second limitation of the “multimenu” is in relation to the fact that the choices of subjects are limited to the words that appear in the matrix. This means that users cannot write everything they want, while this is possible using the classic P300 Speller matrix. In fact, “multimenu” should not be considered for the creation of complex and articulated text messages, but as an easy way to give simple and efficacious tools for communication and/or assistance. As a consequence, it should be modified with respect to the specific user's needs so that it becomes a semantic and significant communicative tool which will best fit the patient's requirements (see Huggins et al., 2011 for discussion). However, precautions should be taken to avoid the risk of a system that is too rigid. If the patient would like to express something that is not in the “multimenu” scheme, the system is sufficiently flexible to give access to the classic P300 Speller from a dedicated submenu.

### Preliminary experiments on end-user subjects

Experiments with the “multimenu” system are actually in progress with two severely impaired patients, representative of the final users of such a system. Patient MB is a 24 years old male affected by ALS, clinically evident since 5 years. He talks and moves on an electronic wheelchair through joystick control. Patient GG is a 55 years old male, who entered a locked-in condition 5 years ago as a consequence of a traumatic injury. He shows preserved cognitive abilities carried out with a simple communication aid which exploits his residual eye movements.

In the experiments composed of 20 EI vs. 20 FC selections with 3 × 3 matrices containing nine Italian words, patient MB reached a communication accuracy of 75%, while GG got an online accuracy of 95%.

These preliminary observations confirm that online accuracy is in the order of those observed in our healthy subjects, even with smaller matrices sizes so indicating that the “multimenu” may be an efficient communication tool. The two pathologies, however, and the dimension of the sample make the observations sporadic and more patients are needed, who will be the object of a future study.

## Conclusions

In conclusion, the present work suggests that a BCI method based on the visually-evoked P300 component can be effectively used to improve the speed of communication. The method uses smaller selection matrices than previous ones and is associated with a reduced time to complete selections of proper items, which are organized into a semantic and hierarchical structure. The combination of high classifier accuracy, high bit rate, and good performance, makes the “multimenu” an effective system for consistent BCI communication (see Wolpaw et al., 2002; Serby et al., 2005 for discussion). As a consequence, this system might represent a useful tool for patients with severe neurodegenerative diseases that affect their communication abilities. It should, however, be mentioned that it has already been stressed that systems which obtain good results in healthy individuals might have a dramatic decrease in accuracy when applied to neurological patients (e.g., Piccione et al., 2006; Silvoni et al., 2009). Nevertheless, some types of patients, such as those affected by Amyotrophic Lateral Sclerosis (ALS), who can use BCI systems with acceptable accuracy (Birbaumer et al., 1999; Kübler et al., 2005), may experience difficulties in communication with classical matrices using “character-by-character” spelling paradigms (e.g., Sellers and Donchin, 2006), even if a regular P300 is normally elicited in an “oddball task.” These patients may have more difficulties when matrices include many items because of uncontrollable eye movements, which increases with the rate of presentation of stimuli, independently of the chosen matrix size (Sellers and Donchin, 2006). Thus, in this and in other pathological conditions, a BCI speller matrix with only a few items and the same or faster speed of selection compared to the conventional P300 speller, might be of help to maintain even a minimal level of communication.

### Conflict of interest statement

The authors declare that the research was conducted in the absence of any commercial or financial relationships that could be construed as a potential conflict of interest.
